# Effects of Estrogen Therapy on the Serotonergic System in an Animal Model of Perimenopause Induced by 4-Vinylcyclohexen Diepoxide (VCD)

**DOI:** 10.1523/ENEURO.0247-17.2017

**Published:** 2018-01-22

**Authors:** Nayara Pestana-Oliveira, Bruna Kalil, Cristiane Mota Leite, Ruither Oliveira Gomes Carolino, Lucas Kniess Debarba, Lucila Leico Kagohara Elias, José Antunes-Rodrigues, Janete A. Anselmo-Franci

**Affiliations:** 1 Department of Physiology, School of Medicine of Ribeirão Preto, University of São Paulo, Ribeirão Preto, São Paulo, Brazil; 2 Department of Morphology, Physiology and Basic Pathology, School of Dentistry of Ribeirão Preto, University of São Paulo, Ribeirão Preto, São Paulo, Brazil

**Keywords:** estrogen, estrogen receptor β, progesterone receptor, dorsal raphe nucleus, amygdala, hippocampus

## Abstract

Chronic exposure to 4-vinylcycloxene diepoxide (VCD) in rodents accelerates the natural process of ovarian follicular atresia modelling perimenopause in women. We investigated why estrogen therapy is beneficial for symptomatic women despite normal or high estrogen levels during perimenopause. Female rats (28 d) were injected daily with VCD or oil for 15 d; 55-65 d after the first injection, pellets of 17β-estradiol or oil were inserted subcutaneously. Around 20 d after, the rats were euthanized (control rats on diestrus and estradiol-treated 21 d after pellets implants). Blood was collected for hormone measurement, the brains were removed and dorsal raphe nucleus (DRN), hippocampus (HPC), and amygdala (AMY) punched out for serotonin (5-HT), estrogen receptor β (ERβ), and progesterone receptor (PR) mRNA level measurements. Another set of rats was perfused for tryptophan hydroxylase (TPH) immunohistochemistry in the DRN. Periestropausal rats exhibited estradiol levels similar to controls and a lower progesterone level, which was restored by estradiol. The DRN of periestropausal rats exhibited lower expression of PR and ERβ mRNA and a lower number of TPH cells. Estradiol restored the ERβ mRNA levels and number of serotonergic cells in the DRN caudal subregion. The 5-HT levels were lower in the AMY and HPC in peristropausal rats, and estradiol treatment increased the 5-HT levels in the HPC and also increased ERβ expression in this area. In conclusion, estradiol may improve perimenopause symptoms by increasing progesterone and boosting serotonin pathway from the caudal DRN to the dorsal HPC potentially through an increment in ERβ expression in the DRN.

## Significance Statement

During the most part of menopausal transition, estradiol fluctuates in a normal range; however, estradiol therapy is effective in attenuating many of perimenopausal symptoms. We demonstrate in a perimenopause animal model that the expression of estrogen receptor β (ERβ) in serotonergic and hippocampal (HPC) neurons, both related to mood disorders, is reduced. Estradiol therapy reverses this deficiency, thus recovering the response of these areas to estrogens. There is also a decline in the number of brain serotonergic neurons and the amount of serotonin in the HPC, which are also reversed by estradiol. Therefore, estrogens therapies that target only β receptors may be an alternative to obtain the beneficial effects of estradiol while eliminating the undesirable side effects of estrogens through α receptors.

## Introduction

At middle age, women progress from the reproductive (premenopause) to non-reproductive (postmenopause) life through a transition period named perimenopause (Harlow et al., 2012). During perimenopause, women may exhibit numerous symptoms, which include variability in cycle length, vasomotor symptoms, dysphoric mood symptoms, insomnia, and somatic symptoms (Mitchell and Woods, 1996; Diaz Brinton, 2012; Brinton et al., 2015). Regarding mood disorders, both epidemiological and clinical studies have consistently demonstrated that after puberty, the risk for depression is higher in women than men, and it reaches the maximum during perimenopause (Deecher et al., 2008).

Ovarian steroids, mainly estradiol and progesterone, affect brain regions involved in the modulation of mood and behavior (McEwen, 1989), and fluctuations in ovarian hormone secretion modify brain neurochemistry (Schmidt et al., 1998; Barth et al., 2015). Moreover, the emotional vulnerability windows that occur throughout women’s lives are correlated with reproductive periods marked by considerable hormonal fluctuations, such as menstruation, pregnancy, postpartum period and perimenopause, thus indicating the pivotal role of sex steroids in the control of affective disorders (Stahl, 2001). The effects of estradiol and progesterone are predominately mediated by their nuclear cognate receptors: estrogen receptors (ERs) and progesterone receptors (PRs; Krege et al., 1998). These receptors are widely expressed in brain regions that control reproduction, as well as regions not typically linked with this function (Shughrue et al., 1997; Brinton et al., 2008; Weiser et al., 2008).

The hormonal profile of perimenopausal women is different from that observed in postmenopause. In postmenopausal women, levels of estrogens are extremely low, whereas in perimenopausal women, during the early and mid-perimenopause levels of estrogens are normal or even elevated compared to the premenopausal period (Reyes et al., 1977; Santoro and Randolph, 2011), and only at late perimenopause period, there is a significant decline on levels of estrogens (Burger et al., 1995; Santoro and Randolph, 2011).

Progesterone levels are clearly lower during perimenopause, which causes a misbalance in the estradiol/progesterone ratio (O’Connor et al., 2009). Considering the high estradiol and low progesterone level scenario, an intuitive treatment would be to supplement progesterone to counterbalance the estradiol dominance. However, there is no consensus about treatment of the symptomatic perimenopausal women and oral contraceptive or estrogen therapies are widely prescribed to relieve perimenopausal symptoms. Estradiol therapy improves several symptoms, such as vasomotor symptoms, thereby improving women’s quality-of-life (Grant et al., 2015). Interestingly, while no correlation has been found between depression and estradiol levels during perimenopause, estrogen therapy is associated with significant improvements in the mood of perimenopausal women with depression symptoms (Schmidt et al., 2000; Soares et al., 2001). Therefore, considering the normal or high levels of estradiol during perimenopause, the use of extra doses of estrogen to ameliorate mood disorders in perimenopausal women is counterintuitive. Thus, to clarify this question, we raise three possibilities: (1) estradiol can increase the expression of PRs (MacLusky and McEwen, 1978; Helena et al., 2009), thus, it is reasonable to suggest that estrogen therapy, by increasing PR expression, compensates for the low plasma levels of progesterone; (2) as estrogen effects on mood predominately occur through ERβ (Bansal and Chopra, 2015; Bastos et al., 2015; Benmansour et al., 2016), estradiol therapy during perimenopause may positively modulate ERβ expression; and (3) because estrogens increases the activity of tryptophan hydroxylase (TPH), the rate-limiting enzyme in serotonin synthesis (Hiroi et al., 2006), it may rectify potential deviations in serotonin synthesis in the dorsal raphe nucleus (DRN), a central nucleus for the control of emotions. To test these hypotheses, we used an animal model of perimenopause in which the natural follicle depletion is accelerated by the 4-vinylcycloxene diepoxide (VCD) retaining residual ovarian tissue (Lohff et al., 2005). Unlike ovariectomized animals, in which the concentrations of ovarian hormones fall abruptly, this ovary-intact animal is suitable to model the natural women progression to perimenopause (Brooks et al., 2016). Thus, the aims of this study were evaluated whether follicular depletion induces alterations on: (1) the plasma concentrations of progesterone; (2) the ERβ and PR mRNA expression in the DRN, dorsal hippocampus (HPC) and amygdala (AMY); (3) the number of TPH-immunoreactive (ir) neurons in the DRN and d) the serotonin content in the dorsal HPC and AMY. Accordingly, we tested whether estradiol therapy can prevent the alterations observed in periestropausal rats.

## Materials and Methods

### Animals

Female Wistar rats at postnatal day (PND)21 were obtained from the animal facilities of the University of São Paulo; rats were housed four per plastic cage (40 × 33 × 17 cm) and maintained on 12/12 h light/dark cycles (lights on at 6:00 A.M.) at a controlled temperature (24 ± 0.5°C). Animals were allowed to acclimate to animal room condition for 7 d before the onset of VCD administration, which started at PND28, as described by Mayer et al. (2002). Food and water were provided *ad libitum*. All procedures were approved by the Committee for Animal Care and Use (2013.1.1412.58.7), University of São Paulo.

### VCD-induced perimenopausal model

It has been shown that the oocyte number in rodents increases markedly toward the end of fetal life, but similar to women, many are lost as they assemble to form primordial follicles. Additionally, postnatal primordial follicles decline significantly for up to two weeks after birth followed by a period of very slow follicle loss, which lasts for several months (Kerr et al., 2013).

An ovary-intact rat model of perimenopause using the chemical, VCD (Mayer et al., 2004; Lohff et al., 2005), has been developed to accelerate the natural process of slow follicular loss. Repeated daily dosing with VCD for 15 d selectively destroys primordial and primary follicles in ovaries of mice and rats by accelerating atresia processes (Springer et al., 1996; Hu et al., 2001; Takai et al., 2003), thus inducing precocious perimenopause/menopause. The advantage of the VCD model over the surgically induced menopausal model (OVX), is that in VCD model ovarian steroids production do not ceases abruptly, as in OVX model, and the residual ovarian tissue produces androgens as in women. In addition, the time of OVX differs substantially among the studies generating conflicting results and misleading conclusions. Lastly, reproductive senescence in aging rodents seems to initiate centrally and not in the ovary as opposed to women (Gore et al., 2000; Kermath and Gore, 2012) therefore, aging rodents are not a good model as well.

The optimal VCD dose was determined as 160 mg/kg during 15 d (Mayer et al., 2004), and the subcutaneous administration route has been described as less harmful to the animals (Reis et al., 2014). This VCD dose was described as toxic only to ovarian follicles when administrated to juvenile rats (one-month-old), while in older rats (three-month-old), this same dose induced toxicity to other organs besides ovaries (Frye et al., 2012). Moreover, the administration of VCD at PND28 has the advantage to dissociate the effects of follicular depletion from those of aging by the time of the experiment when the rats are in periestropause but still young (around three to four months of age; Mayer et al., 2005; Van Kempen et al., 2011). It is important to note that this advantage is at the same time a limitation of the model. In one hand, the model allows to isolate the effects of follicular depletion from aging. In the other hand, the natural process of follicular depletion progress in parallel with aging, thus the resultant symptoms experienced during perimenopause can also result from of the impact of follicular depletion over aging and vice versa. Therefore, although ovary-intact model of perimenopause induced by VCD exposure in juvenile female rats is currently considered the model that more closely approximates to the natural human progression to menopause (Van Kempen et al., 2011), the interpretation of the results must be carefully addressed.

### Experimental design

**Figure 1.**
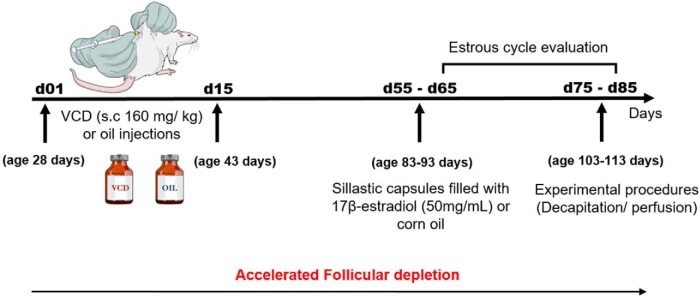
Schematic diagram showing the timeline of the experimental protocol.

Peripubertal female rats at PND28 were injected daily with VCD (Sigma-Aldrich) subcutaneously administered at a dose of 160 mg/kg (128 mg/ml diluted in corn oil; 1.25 ml/kg body weight) for 15 d. Corn oil (O; 1.25 ml/kg of body weight) was used in the control rats. Estrous cycle was monitored from day 65-85 after the onset of VCD treatment. Since the percentage of irregular cycle in our colony is around 20% in both O + O and VCD + O groups, only rats cycling regularly were used in the experiment. Around 55-65 d after the first injection, pellets of 17β-estradiol (E) or O were implanted subcutaneously (groups O + O; VCD + O; VCD + E). Rats O + O and VCD + O were decapitated between 75 and 85 d after the onset of VCD/Oil treatment between 9:00 and 11:00 A.M. of diestrus. VCD + E rats were decapitated exactly 21 d after the onset of E therapy, regardless the phase of the estrous cycle, at the same time of the day. Following decapitation, trunk blood was collected for estradiol and progesterone measurements, the brain was removed, and the dorsal HPC and AMY were punched out to assess the 5-HT content by HPLC/ED and ERβ and PR mRNA levels. The DRN was dissected to analyze the ERβ and PR mRNA levels. Another set of rats under the same experimental protocol was euthanized via perfusion for the immunohistochemical evaluation of TPH-positive cells in the DRN Fig. 1. 

### Estrogen therapy

Sillastic capsules filled with 8-μl 17β-estradiol 50 mg/ml (Sigma; group VCD + E) or oil (groups O + O and VCD + O) were subcutaneously implanted into the dorso-lateral region of VCD/oil female rats under anesthesia (55 mg/kg ketamine, Agener; and 10 mg/kg xylazine, Coopers of Brazil; s.c.). Pellets were prepared as described previously (Kiss et al., 2012). After surgery, the animals received prophylactic antibiotic (Pentabiotico Fort Dodge; 0.2 ml/rat, i.m.) and antiinflammatory treatment (Banamine, Schering-Plow; 2.5 mg/kg, s.c.).

### Hormonal assays

Trunk blood from decapitation was collected into heparinized tubes, and the plasma was stored frozen (-20°C) for hormone assays. The plasma estradiol concentrations were determined via an ELISA estradiol kit (EIA 2693; DRG Instruments GmbH). The progesterone concentrations were determined by radioimmunoassay (RIA) using specific kits provided by MP Biomedicals. The intraassay coefficients of variation were 4.7% and 3.6%, and the lower limits of detection were 8.6 pg/ml and 0.02 ng/ml for estradiol and progesterone, respectively. All samples were measured in duplicate. To avoid interassay variation, all samples from the same experiment were measured in the same assay.

### Brain microdissections

After decapitation, the brains were rapidly removed and frozen at -70°C. Microdissections were obtained according to Palkovits (1973). The HPC and AMY were microdissected to assess the serotonin content, and DRN was microdissected to analyze the ERβ and PR mRNA expression. Thick coronal brain sections were cut in a cryostat at -15°C. For the AMY and dorsal HPC, a 1500-µm section was obtained starting at approximately -1.92 mm from bregma (Paxinos and Watson, 2007) and mounted onto chilled glass slides for microdissection using the punch technique (Palkovits, 1973). The AMY and dorsal HPC were dissected with 1.5- and 1.0-mm diameter needles, respectively. The DRN was dissected from one section of 1000 µm, from -7.2 mm from bregma (Paxinos and Watson, 2007), in one punch of each side of the brain with a 0.7-mm diameter needle.

### Serotonin content measurements (HPLC/ED)

The 5-HT content was determined by HPLC coupled to an electrochemical detector. Briefly, microdissections of the dorsal HPC and AMY were individually homogenized in 100 µl of a solution that contained 0.15 M perchloric acid, 0.1 mM EDTA and 380 nM isoproterenol (ISOP; internal standard). Each homogenate was centrifuged at 15,700 × *g* (for 5 min at 4°C); the supernatants were filtered through a 0.22 µM membrane (Durapore, Millipore) and subsequently injected into an HPLC system with an autoinjector (SIL-10ADVP; Shimadzu). The separation was performed at 32°C in a reverse phase column 250 × 4 mm (Kinetex EVO C18, 5 µm; Phenomenex), preceded by a 4 × 4 mm precolumn (Kinetex EVO C18, 5 µm; Phenomenex). The mobile phase was composed of 75 mM sodium dihydrogen phosphate, 10 mM sodium chloride, 25 µM EDTA, 1.7 mM sodium 1-octansulfonic acid (Sigma-Aldrich), and 4% methanol (Merck Chemical Inc.). The pH was adjusted to 3.0 with phosphoric acid. The flow rate of the pump (LC-10ADVP; Shimadzu) was 0.8 ml/min, and the detector potential was 0.65 V (Decade; Antec). Each sample produced a chromatogram, which was recorded and analyzed with the software Class-VP (Shimadzu). 5-HT and ISOP were identified by their peak retention time and quantified by an internal standard method based on their peak height. All samples of each brain area were processed in the same analysis to avoid interassay variation. In the dorsal HPC and AMY, the intraassay variation was 6.1% and 5.0%, respectively. For each sample, the 5-HT level was normalized to the protein content and expressed as picograms per microgram of protein (pg/μg).

### Quantitative real-time PCR

Total RNA was isolated from the AMY and dorsal HPC samples using TRIzol reagent (Invitrogen) according to the manufacturer’s protocol. The RNA concentrations were determined using a Nanodrop 2000c UV-Vis Spectrophotometer (Thermo Scientific). Quantitative real-time PCR for ERβ and PR were performed using a Step One Plus real-time PCR system purchased from Applied Biosystems. The TaqMan Genes Expression Assay used in this study was Rn00562610_m1 for ERβ (gene symbol: *Ers2*) and Rn01448227_m1 for PR (gene symbol: *Pgr*). Each PCR reaction was performed in triplicate. Water (instead of cDNA) was used as a negative control. The housekeeping genes for normalizing ERβ and PR expression included β-actin (Rn00667869_m1). The determination of the gene transcript levels in each sample was obtained by the ΔΔCT method. For each sample, the threshold cycle (Ct) was determined and normalized to the average of the housekeeping gene (ΔCt = CtUnknown - CtHousekeeping gene). The relative mRNA level in the unknown sample relative to the calibrator group (O + O group) was calculated as 2^-ΔΔCt^, where ΔΔCt = ΔCtUnknown - ΔCtCalibrator (Livak and Schmittgen, 2001).

### Perfusion and immunohistochemistry for TPH

Rats were deeply anesthetized with ketamine (ketamine hydrochloride, Agner; 106 mg/kg) and xylazine (xilasina, Coopazine, Coopers; 18.6 mg/kg) and transcardially perfused with PBS, followed by ice-cold 4% paraformaldehyde. Serial coronal sections of 30 µm were cut in four series that represented the antero-posterior length of the DRN. The immunolabeling of TPH was performed on free-floating sections 120 μm apart. The sections were rinsed at room temperature in 0.01 M PBS (pH 7.3, 3 × 10 min), incubated for 10 min in 1% hydrogen peroxide, and rinsed again in 0.01 M PBS (5 × 10 min). The sections were subsequently incubated for 60 min at room temperature in PBS buffer that contained 1% BSA (Sigma Chemical Co) to block non-specific binding. The sections were incubated for 40 h at 4°C with sheep anti-TPH polyclonal antibody (1:2000; Millipore) and then washed in 0.01 M PBS (5 × 5 min) at room temperature. The sections were subsequently incubated with the biotinylated rabbit anti-sheep IgG (1:600; Vector Laboratories) for 1 h at room temperature. Signal amplification was performed using an avidin-biotin kit (1:100; Vector Laboratories) for 1 h at room temperature. A solution of 25 mg/ml nickel sulfate, 0.2 mg/ml 3,3’-diaminobenzidine-HCl (DAB) and 0.03% H_2_O_2_ (Ni-DAB) diluted in 0.175 M sodium acetate was used as the chromogen. The sections were mounted on slides (Fisherbrand Superfrost Plus; Fisher Scientific) treated with subbing solution (0.1% gelatin and 0.01% chromium potassium sulfate), allowed to dry in the dark, coverslipped with Entellan (Merck).

### Microscopy

Brightfield imaging of TPH-ir neurons was performed using a Zeiss Axioskope 2 plus microscope. The number of TPH-ir neurons was quantified throughout the rostro-caudal extent of the DRN from bregma −7.32 to −8.58 mm. The number of TPH-positive cells was analyzed at three anatomic levels, rostral (−7.32 mm from bregma), mid (−7.80 mm from bregma) and caudal (−8.5 mm from bregma), each of which was divided into three subregions, including lateral, dorsal, and ventral regions, as previously described (Kunimura et al., 2015). Two sections from each level per animal were analyzed. The subsectional analyses of the DRN were performed on the basis of neuroanatomical data showing differential projection from distinct subregions of the DRN to a number of brain areas (Lowry, 2002; Michelsen et al., 2007). Digital images were subsequently converted to a tagged image file format and imported into Adobe Photoshop (Adobe Photoshop Lightroom, version 5.3; Adobe Systems, Inc.), in which the color balance was generally adjusted for presentation.

### Statistical analysis

Except for gene expression, all comparisons were performed using one-way ANOVA followed by Newman–Keuls *post hoc* test. For gene expression, the comparison was performed in relation to the control group (O + O group) using one-way ANOVA followed by Fisher’s LSD test. Data are presented as the mean ± SEM. Significance was accepted at *p* ≤ 0.05. All statistical analyses and graphs were performed using GraphPad Prism software (GraphPad Software).

## Results

**Figure 2.**
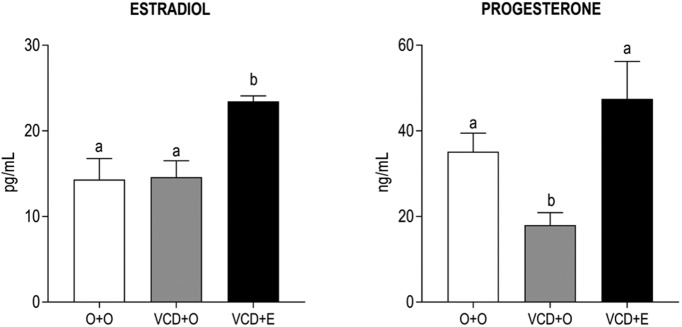
Effects of follicular depletion induced by VCD and chronic 17β-estradiol (E) treatment on estradiol (left panel) and progesterone (right panel) plasma concentrations. Female rats at 28 d of age were subcutaneously injected with corn oil (O) or VCD for 15 consecutive days. Fifty-five days after the first injection of VCD/oil, rats received a subcutaneous capsule that contained corn oil (groups O + O and VCD + O) or 17β-estradiol (VCD + E). Rats were decapitated during the diestrous phase (O + O and VCD + O groups) or 21 d after the onset of E therapy (VCD + E), from 75 to 85 d after the onset of VCD/oil administration, between 9:00 and 11:00 A.M. Data are presented as the mean ± SEM. Significance was defined as *p* < 0.05. Different letters indicate significant differences (*n* = 6-9).

The estradiol levels in the VCD + O group did not vary compared with the O + O group. As expected, after 21 d of 17β-estradiol therapy, the estradiol plasma concentrations were significantly higher in the VCD + E animals than the other groups (Fig. 2, left panel; *p* < 0.001). In the VCD + O rats, plasma progesterone concentrations were significantly lower than in the O + O group (*p* < 0.001), and estradiol treatment restored it to the control levels (Fig. 2, right panel).

**Figure 3.**
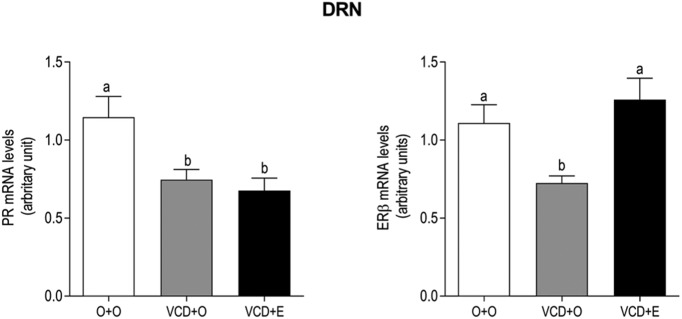
Effects of follicular depletion induced by VCD and chronic 17β-estradiol (E) administration on the relative mRNA expression of PR (left panel) and ERβ (right panel) in the DRN. Female rats at 28 d of age were subcutaneously injected with corn oil (O) or VCD for 15 consecutive days. Fifty-five days after the first injection of VCD/oil, rats received a subcutaneous capsule that contained corn oil (groups O + O and VCD + O) or 17β-estradiol (VCD + E). Rats were decapitated during the diestrous phase (O + O and VCD + O groups) or 21 d after the onset of E therapy (VCD + E), from 75 to 85 d after the onset of VCD/oil administration, between 9:00 and 11:00 A.M. (*n* = 6-9). Data are presented as the mean ± SEM. Significance was accepted at *p* < 0.05.

**Figure 4.**
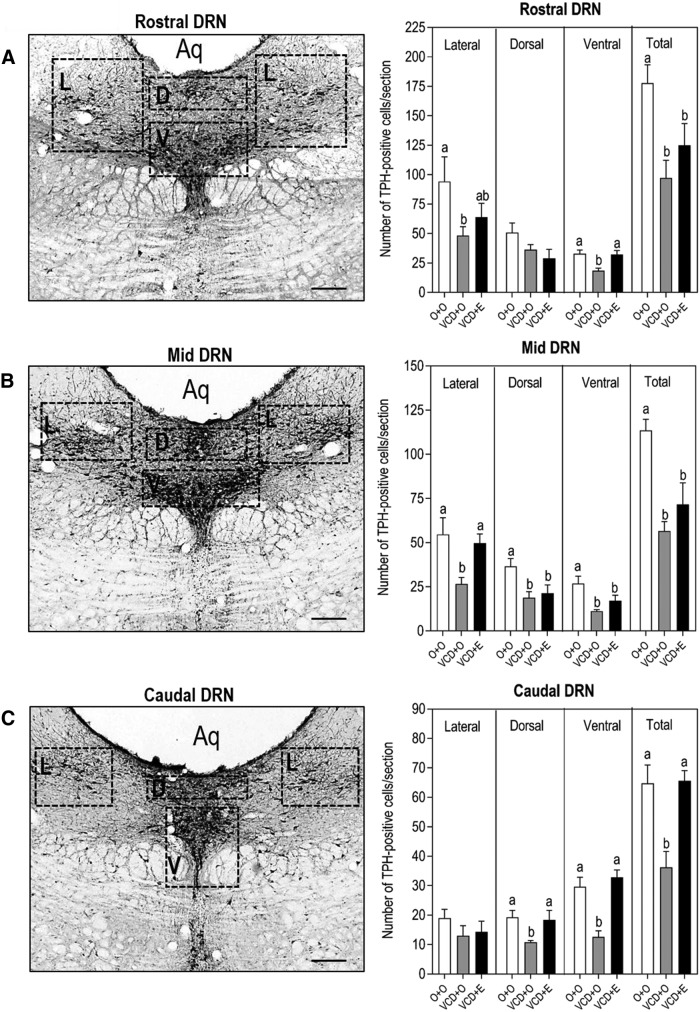
Effect of follicular depletion induced by VCD and chronic 17β-estradiol (E) administration on TPH immunoreactivity in the DRN. Representative photomicrographies (10×) of coronal sections of the DRN immunostained for TPH in rostral (***A***; -7.32 mm from bregma), mid (***B***; -7.80 mm from bregma), and caudal (***C***; -8.28 mm from bregma) sections of the DRN. Dotted boxes indicate the lateral (L), dorsal (D), and ventral (V) subregions at each level of the DRN. Aq, aqueduct. Scale bar: 50 µm. Bargraphs show the number of TPH-positive cells per section in the rostral (top panel), mid (mid panel), and caudal (lower panel) sections of the DRN. Female rats at 28 d of age were subcutaneously injected with corn oil (O) or VCD for 15 consecutive days. Fifty-five days after the first injection of VCD/oil, rats received a subcutaneous capsule that contained corn oil (groups O + O and VCD + O) or 17β-estradiol (VCD + E). Rats were perfused during the diestrous phase (O + O and VCD + O groups) or 21 d after the onset of E therapy (VCD + E), from 75 to 85 d after the onset of VCD/oil administration, between 9:00 and 11:00 A.M. These photomicrographies represent sections of a O + O rat. Data are presented as the mean ± SEM. Significance was accepted at *p* < 0.05. Different letters indicate significant differences (*n* = 4-6).

In the DRN, the PR mRNA expression in the VCD + O was lower than the control rats (*p* = 0.0156), and this effect was not reversed by estradiol treatment (Fig. 3, left panel). Similarly, the rats in periestropause also expressed less mRNA for ERβ (*p =* 0.0409); however, estradiol treatment was effective in reversing this effect (Fig. 3, right panel). As expected, immunoreactive TPH cells were identified throughout the rostral-caudal length of the DRN from bregma −7.32 to −8.5 mm (Fig. 4*A–C*). The overall effect on TPH-positive cells in the entire DRN showed a decreased number of TPH-positive cells/section in VCD-treated animals compared to the control (*p =* 0.0062) that was partially restored by estradiol therapy (O + O 119.3 ± 12.48; VCD + O 67.73 ± 9.107; VCD + E 90.63 ± 11.23; Table 1). The number of TPH-positive cells was also analyzed separately at three anatomic levels, rostral, mid, and caudal, each of which was divided into three subregions, including the lateral, dorsal, and ventral regions (Fig. 4*A–C*). At the rostral part (Fig. 4, top bargraph panel), the number of TPH-positive cells in the VCD + O group was lower than in the O + O group in the lateral and ventral parts of the DRN (*p* < 0.05). Estradiol therapy (VCD + E) was able to restore the number of TPH-positive cells only in the ventral subregion (*p* < 0.001). On the other hand, estradiol effect on rostral-ventral subregion was not sufficient to reestablish the total amount of TPH-positive cells identified in the O + O group. At the mid-level (Fig. 4, mid bargraph panel), the number of TPH-positive cells in the VCD + O group was lower than in the O + O group in all subregions (*p* < 0.05) and estradiol therapy was able to restore the number of TPH-positive cells only in the lateral subregion (*p* < 0.05). Overall, estradiol treatment appeared to be ineffective in restoring the number of TPH cells at the mid-level of the DRN as demonstrated by the comparison of the total number of TPH-positive cells in the VCD + E group versus the VCD + O group. At the caudal level (Fig. 4, lower bargraph panel), the lateral subregion was not affected by VCD and estradiol therapy. On the other hand, the number of TPH-positive cells was substantially decreased in the VCD + O group in the dorsal and ventral subregions (*p* < 0.05 and *p* < 0.001, respectively); estradiol therapy was effective in reverting this effect in both subregions when analyzed individually (*p* < 0.05 for caudal dorsal and *p* < 0.001 for caudal-ventral subregions), as well as in the entire caudal level of the DRN when all subregions were analyzed collectively (total; *p* < 0.001).

**Table 1.** Summary of estradiol therapy effects in the perimenopausal rat model

Parameters	VCD + O	VCD + E
Estradiol plasma concentration, pg/ml	=	↑
Progesterone plasma concentration, ng/ml	↓	=
PR mRNA levels in DRN, arbitrary units	↓	↓
ERβ mRNA levels in DRN, arbitrary units	↓	=
Number of TPH-ir cells in total DRN	↓	=
PR mRNA levels in AMY, arbitrary units	=	=
ERβ mRNA levels in AMY, arbitrary units	=	=
5-HT content in AMY, pg/µg protein	↓	↓
PR mRNA levels in HPC, arbitrary units	=	=
ERβ mRNA levels in HPC, arbitrary units	=	↑
5-HT content in HPC, pg/µg protein	↓	=

All parameters were evaluated in the VCD + O and VCD + E groups in relation to the control (O + O).

**Figure 5.**
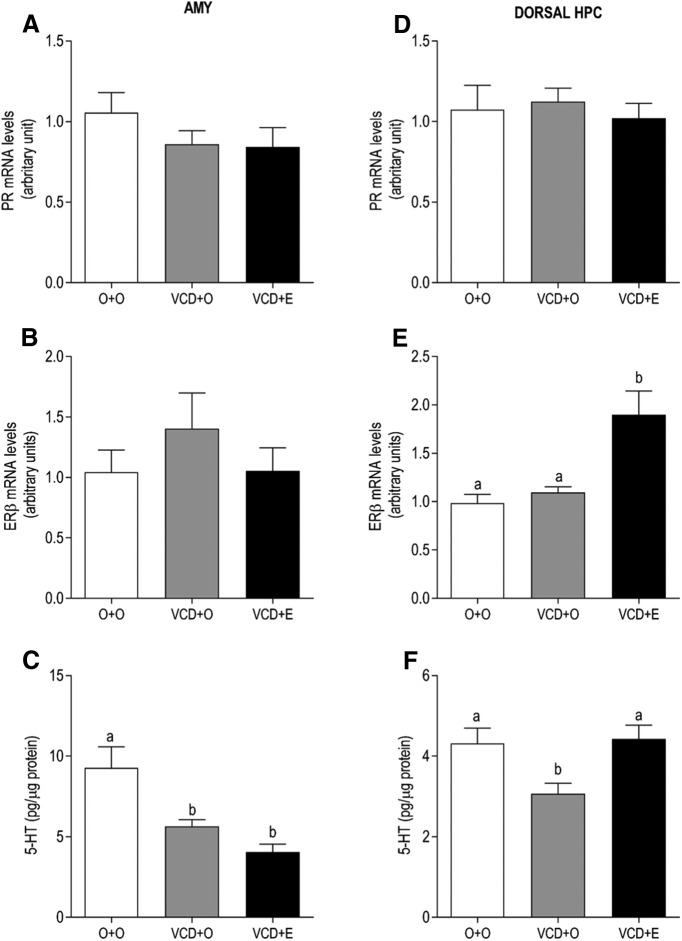
Effects of follicular depletion induced by VCD and chronic 17β-estradiol (E) administration on PR and ERβ mRNA levels and 5-HT content in the AMY (***A–C***) and dorsal HPC (***D–F***). Female rats at 28 d of age were subcutaneously injected with corn oil (O) or VCD for 15 consecutive days. Fifty-five days after the first injection of VCD/oil, rats received a subcutaneous capsule that contained corn oil (groups O + O and VCD + O) or 17β-estradiol (VCD + E). Rats were decapitated during the diestrous phase (O + O and VCD + O groups) or 21 d after the onset of E therapy (VCD + E), from 75 to 85 d after the onset of VCD/oil administration, between 9:00 and 11:00 A.M. Data are presented as the mean ± SEM. Significance was accepted at *p* < 0.05. Different letters indicate significant differences (*n* = 6-9).

In the AMY and dorsal HPC, the levels of PR mRNA remained unaltered in all groups (Fig. 5*A*,*D*, respectively). The levels of ERβ mRNA in the dorsal HPC was higher in the VCD + E group than the O + O and VCD + O groups (Fig. 5*E*; *p* = 0.0012), while in AMY, the levels of ERβ mRNA remained unaltered in all groups (Fig. 5*B*). Regarding 5-HT, the VCD + O rats exhibited decreased 5-HT in both areas, dorsal HPC (Fig. 5*F*; *p* < 0.05) and AMY (Fig. 5*C*; *p* < 0.05). However, estradiol therapy was effective in restoring 5-HT levels only in the dorsal HPC (Fig. 5*F*; *p* < 0.05 vs O + O and VCD + O groups). The data are summarized in Table 1, and the statistical analyses shown in detail in Table 2.

**Table 2.** Statistical analyses performed in all experiments

Figure	Data structure	Type of test	Statistical results	
2, left panel	Normal distribution	Ordinary one-way ANOVANewman–Keuls *post hoc* test	*F*_(2,18)_ = 10.06	**/p**= 0.0012
		O+O vs VCD+OO+O vs VCD+EVCD+O vs VCD+E		*p >* 0.05 *p <* 0.001 *p <* 0.001
2, right panel	Normal distribution	Ordinary one-way ANOVANewman–Keuls *post hoc* test	*F*_(2,24)_ = 7.237	**/p**= 0.0035
		O+O vs VCD+OO+O vs VCD+EVCD+O vs VCD+E		*p <* 0.05 *p >* 0.05 *p <* 0.001
3, left panel	Normal distribution	Ordinary one-way ANOVAFisher's LSD*post hoc* test	*F*_(2,15)_ = 6.74295.00% CI	**/p**= 0.0082
		O+O vs VCD+OO+O vs VCD+E	0.0873 to 0.7120.1827 to 0.757	*/p =* 0.0156 */p =* 0.0033
3, right panel	Normal distribution	Ordinary one-way ANOVAFisher's LSD*post hoc* test	*F*_(2,16)_ = 4.92195.00% CI	**/p**= 0.0216
		O+O vs VCD+OO+O vs VCD+E	0.0179 to 0.751-0.485 to 0.184	*/p =* 0.0409 */p =* 0.3540
4*A*	Normal distribution	Ordinary one-way ANOVANewman–Keuls *post hoc* test			
		Rostral-lateral DRN O+O vs VCD+OO+O vs VCD+EVCD+O vs VCD+E Rostral-dorsal DRN O+O vs VCD+OO+O vs VCD+EVCD+O vs VCD+E Rostral-Ventral DRN O+O vs VCD+OO+O vs VCD+EVCD+O vs VCD+E Rostral-Total DRN O+O vs VCD+OO+O vs VCD+EVCD+O vs VCD+E	*F*_(2,14)_ = 4.363 *F*_(2,13)_ = 1.445 *F*_(2,15)_ = 7.104 *F*_(2,15)_ = 6.304	*/p =* 0.0337 *p <* 0.05 *p >* 0.05 *p >* 0.05 */p =* 0.2712 *p >* 0.05 *p >* 0.05 *p >* 0.05 */p =* 0.0068 *p <* 0.05 *p >* 0.05 *p <* 0.001 */p =* 0.0103 *p <* 0.001 *p <* 0.05 *p >* 0.05
4*B*	Normal distribution	Ordinary one-way ANOVANewman–Keuls *post hoc* test			
		Mid-lateral DRN O+O vs VCD+OO+O vs VCD+EVCD+O vs VCD+E Mid-dorsal DRN O+O vs VCD+OO+O vs VCD+EVCD+O vs VCD+E Midl-Ventral DRN O+O vs VCD+OO+O vs VCD+EVCD+O vs VCD+E Mid-Total DRN O+O vs VCD+OO+O vs VCD+EVCD+O vs VCD+E	*F*_(2,13)_ = 5.039 *F*_(2,15)_ = 4.992 *F*_(2,14)_ = 7.030 *F*_(2,14)_ = 13.30	*/p =* 0.0240 *p <* 0.05 *p >* 0.05 *p <* 0.05 */p =* 0.0218 *p <* 0.05 *p <* 0.05 *p >* 0.05 */p =* 0.0077 *p <* 0.001 *p <* 0.05 *p >* 0.05 */p =* 0.0006 *p <* 0.0001 *p <* 0.001 *p >* 0.05

4*C*	Normal distribution	Ordinary one-way ANOVANewman–Keuls *post hoc* test			
		Caudal-lateral DRN O+O vs VCD+OO+O vs VCD+EVCD+O vs VCD+E Caudal-dorsal DRN O+O vs VCD+OO+O vs VCD+EVCD+O vs VCD+E Caudal-Ventral DRN O+O vs VCD+OO+O vs VCD+EVCD+O vs VCD+E Caudal-Total DRN O+O vs VCD+OO+O vs VCD+EVCD+O vs VCD+E	*F*_(2,11)_ = 0.994 *F*_(2,10)_ = 3.956 *F*_(2,11)_ = 12.61 *F*_(2,9)_ = 9.990	*p* = 0.4009 *p >* 0.05 *p >* 0.05 *p >* 0.05 */p =* 0.05 *p <* 0.05 *p >* 0.05 *p <* 0.05 */p =* 0.0014 *p <* 0.001 *p >* 0.05 *p <* 0.001 */p =* 0.0052 *p <* 0.001 *p >* 0.05 *p <* 0.05
5*A*	Normal distribution	Ordinary one-way ANOVAFisher's LSD *post hoc* test	*F*_(2,23)_ = 1.17295.00% Cl	**/p**= 0.3276
		O+O vs VCD+OO+O vs VCD+E	-0.164 to 0.5585-0.1589 to 0.5858	*/p =* 0.3476 */p =* 0.3157
5*B*	Normal distribution	Ordinary one-way ANOVAFisher's LSD *post hoc* test	*F*_(2,21)_ = 0.807895.00% CI	**/p**= 0.4592
		O+O vs VCD+OO+O vs VCD+E	-1.125 to 0.4039-0.7755 to 0.7531	*/p =* 0.4415 */p =* 0.9991
5*C*	Normal distribution	Ordinary one-way ANOVANewman–Keuls *post hoc* test	*F*_(2,20)_ = 9.697	**/p**= 0.0011
		O+O vs VCD+OO+O vs VCD+EVCD+O vs VCD+E		*p <* 0.001 *p <* 0.0001 *p >* 0.05
5*D*	Normal distribution	Ordinary one-way ANOVAFisher's LSD *post hoc* test	*F*_(2,21)_ = 0.207395.00% CI	**/p**= 0.8144
		O+O vs VCD+OO+O vs VCD+E	-0.4267 to 0.3285-0.3241 to 0.431	*/p =* 0.9333 */p =* 0.9216
5*E*	Normal distribution	Ordinary one-way ANOVAFisher's LSD *post hoc* test	*F*_(2,16)_ = 10.9595.00% CI	**/p**= 0.0010
		O+O vs VCD+OO+O vs VCD+E	-0.6103 to 0.3933-1.433 to -0.3916	*/p =* 0.8228 */p =* 0.0012
5*F*	Normal distribution	Ordinary one-way ANOVANewman–Keuls *post hoc* test	*F*_(2,16)_ = 4.797	**/p**= 0.0233
		O+O vs VCD+OO+O vs VCD+EVCD+O vs VCD+E		*p <* 0.05 *p >* 0.05 *p <* 0.05

## Discussion

The current clinical practice to ameliorate perimenopausal symptoms relies on the use of estradiol therapy, although perimenopausal women are not estradiol deficient. The present study contributes to the understanding of this paradox by providing evidence that estradiol therapy appears to improve perimenopause symptoms, at least in part, by increasing the biosynthesis of progesterone and boosting the serotonin pathway from the caudal DRN to the dorsal HPC potentially through an increment in ERβ expression in the DRN. The ability to upregulate ERβ expression appears to be the estradiol key function to rectify the impairments in the serotonergic system induced by ovarian follicle depletion.

In the perimenopause animal model induced by VCD, we have recently shown that the ovarian hormones changes are similar to those exhibited by women in perimenopause, i.e., estradiol plasma concentrations remain unchanged, whereas progesterone plasma concentrations are low (Reis et al., 2014). These data were confirmed in the present study, which indicated no changes in the estradiol levels and low levels of progesterone in periestropausal rats. The increased levels of estradiol in the estradiol-treated rats confirm the efficiency of treatment. Thus, since estradiol levels remain normal, many of the symptoms experienced by women during perimenopause might be ascribed to the low levels of progesterone. However, if estradiol therapy is initiated before the establishment of menopause, symptoms, such as hot flashes and mood disorders, are reduced (Wiklund et al., 1993; Morgan et al., 2007), which indicates that important changes in the brain biochemistry may occur as a result of ovarian senescence and estradiol signaling in the brain is somehow impaired. In this prism, the neurocircuitry responsive to estradiol becomes refractory, and exogenous estradiol would be necessary to reestablish the normal function.

Because progesterone levels were restored in response to estradiol therapy in periestropausal rats, it is reasonable to suppose that some of the estradiol beneficial effects appear to occur by adjusting progesterone levels. The mechanisms by which estradiol increases progesterone remain unclear; however, it has been shown that estradiol can modify steroidogenesis in the ovaries. In female rats, estradiol increases the activity of the enzyme 3-β-hydroxysteroid dehydrogenase-isomerase (3-βHSD) that catalyzes the conversion of pregnenolone to progesterone and reduces the activity of the enzyme 17-hydroxylase (17-OH), which cleaves progesterone to 17-OH-progesterone (Munabi et al., 1983). Thus, by increasing synthesis and decreasing catabolism of progesterone, estradiol may induce an increase in the plasma progesterone concentrations. In addition, the production of progesterone in a hypothalamic astrocyte culture is also increased by estradiol (Micevych et al., 2008). Therefore, the peripheral and central progesterone may be increased in VCD rats treated with estradiol therapy.

Recent studies have established a relationship between perimenopausal symptoms and neuroprogestins, such as allopregnanolone (ALLO), a progesterone metabolite, which exerts anxiolytic effects by acting as an agonist on the GABA_A_ (Lovick, 2006). Thus, it is possible that the beneficial effects of estradiol therapy during postmenopause occur through ALLO and progesterone. In accordance, low progesterone levels have been associated with mood swings that occur not only in perimenopause but also premenstrual syndrome and postpartum depression (Angst et al., 2001; Lovick, 2012; Lovick et al., 2017). Animal data have shown that progesterone reduces both anxiety and depressive behaviors in rodents (Mora et al., 1996; Walf et al., 2004; Frye, 2011), and as luteal progesterone drops during diestrus, rats exhibit anxiety-like behaviors, which are enhanced during periestropause (Reis et al., 2014). Altogether, these data suggest that low progesterone or increased estradiol to progesterone ratio might be involved in the development of some psychological symptoms in perimenopausal women.

As one of the major actions of estradiol is to induce PR expression in many regions of the CNS, including serotonergic neurons (MacLusky and McEwen, 1978; Bethea, 1994; Alves et al., 2000; Helena et al., 2009; Furuta et al., 2010) we hypothesized that estradiol could increase PR expression in the DRN because the effects of estradiol on the regulation of affective disorders appear to be, in part, through the serotonergic system. However, we have not found an increase on PR mRNA levels in any of the brain area evaluated in the present study. There are some important differences in our study in relation to previous works that might be generating conflicting results. Our results from periestropausal rats may be different from those of ovariectomized animals since their hormonal profile are different (Frye et al., 2012). Moreover, VCD-treated rats are still cycling, and it is known that PR regulation changes according to the phase of estrous cycle (Guerra-Araiza et al., 2000; Sá and Fonseca, 2017). In addition, PR regulation by estradiol changes with the reproductive status, region of the brain and age (Quadros and Wagner, 2008; Grieb et al., 2017). Finally, since it was shown that PR expression in response to estradiol is attenuated in old female rats compared to young rats (Furuta et al., 2010), we may hypothesize that the low PR expression in the periestropausal rats even with normal levels of estradiol represent a decrease on serotonergic neurons sensitivity to positive estradiol action, consistent with reproductive aging.

It has been postulated that ERα signaling is closely related to reproductive function, whereas ERβ signaling is relevant for non-reproductive functions, such as learning, memory and affective behavior (Rissman et al., 2002; Walf, 2010). In the DRN, ERβ is the predominant ER isoform, wherein 90% of the ERβ-ir neurons co-express TPH (Mitra et al., 2003; Nomura et al., 2005; Suzuki et al., 2013). Therefore, we investigated the expression of ERβ mRNA in the DRN of periestropausal rats. Our data showed that these rats exhibit lower expression of ERβ mRNA in the DRN compared with the control rats. Although there are no data regarding ERβ in perimenopause, it has been shown in mice and rats that there is a generalized age-related decrease in the ERβ levels in the cerebral cortex, HPC, olfactory bulb, AMY, and raphe nucleus, as well as other areas (Mehra et al., 2005; Sharma and Thakur, 2006; Yamaguchi-Shima and Yuri, 2007). However, in these studies, the comparison was made between young and middle-aged or old females, which makes it difficult to distinguish whether this decrease in ERβ is a result of aging or ovarian senescence. In contrast, in the present study, all rats were around four months old; thus, the aging factor can be excluded. Moreover, it has been shown that ovariectomy induces a decrease in ERβ-positive cells in the DRN, which is reversed by an ERβ agonist (Suzuki et al., 2013). Our data indicated that the decrease of ERβ mRNA in the DRN of periestropausal rats was reversed by estradiol. This upregulation of ERβ induced by estradiol appears to be exerted through ERβ activation, because it has been shown that the selective ERβ agonist LY3201 increases ERβ expression in the DRN (Suzuki et al., 2013). It is well established that estradiol activation of ERβ signaling exerts antidepressant effects as a result of its excitatory actions on serotonergic neurons, which include increasing serotonin synthesis in the DRN (Lu et al., 1999; Bethea et al., 2000; Gundlah et al., 2005; Hiroi et al., 2006) and decreasing serotonin degradation by inhibiting monoamino-oxidase activity, thus increasing serotonin availability in the synaptic clefts (Luine and Rhodes, 1983; Ortega-Corona et al., 1994; Smith et al., 2004; Gundlah et al., 2005; Osterlund, 2010). These actions and the increase in the firing rate of DRN neurons (Robichaud and Debonnel, 2005) induced by estradiol appear to be, at least in part, a result of the up regulation of ERβ. Thus, in addition to the low progesterone levels, the decrease in estradiol signaling through ERβ in the DRN may account for the development of some perimenopausal symptoms. The DRN is composed of distinct subregions that project to different sites of the brain to control in an independent, yet integrative manner different physiologic and behavioral processes (Graeff et al., 1996). In the present study, most subregions in the rostral, mid, and caudal DRN of periestropausal rats exhibited a reduced number of TPH-ir neurons associated with the decrease in the ERβ mRNA levels in this nucleus. Nevertheless, estradiol therapy was able to selectively increase the number of TPH-ir neurons only in a few subregions of the DRN related to the control of stress-related brain areas and emotional behaviors, such as the rostral-ventral DRN, the mid-lateral DRN and caudal dorsal-ventral DRN. It is not possible to correlate with certainty functional properties based only in the topographical distribution of serotonergic neurons within the nucleus. However, electrophysiological studies in behaving animals have provided important findings that indicate functional specialization of topographically organized subpopulations of serotonergic neurons (for review, see Lowry, 2002). The physiologic relevance of the increase of TPH-ir cells induced by estradiol in the mid-lateral DRN is unclear. However, it has been shown that the lateral wings of the mid DRN send projections to the arcuate and ventromedial nuclei of the hypothalamus and the lateral and ventral posterior nuclei of the thalamus and may be involved in the regulation of many physiologic processes (Monti, 2010).

The HPC and the AMY are the main limbic structures targeted by DRN efferent projections. The mid-dorsal DRN sends out collaterals to the basolateral and central AMY (Lowry, 2002), potentially to regulate anxiety-related behavior. The subregion analyses of the DRN revealed genuine topographical differences on serotonergic response to estradiol. It has been shown previously that distinct subregions of the DRN projects to different target areas in the brain (Michelsen et al., 2007). Together, the differential projections and responsiveness to estradiol may explain many of the paradoxical effects of estradiol in the brain. The sole analysis of estradiol effect in total DRN seems to cover estradiol selective modulation of DRN serotonergic neurons. The mechanism by which estradiol selectively upregulates the number of serotonergic neurons in DRN subregions is unknown. However, the existence of subpopulations of serotonergic neurons that differ in morphology, phenotype and receptor type warrants further investigation.

In periestropausal rats, TPH-ir neurons were reduced in the mid-dorsal DRN in association with a reduction in the serotonin content in the AMY. Estradiol therapy was unable to restore the number of TPH-ir neurons in the mid-dorsal DRN or the serotonin content in the AMY. Because no changes were identified in the expression of PR and ERβ, the mid-dorsal DRN/AMY serotonergic circuit appears to be regulated by a factor other than directly by the ovarian steroids.

The serotonergic fibers that originate from the caudal DRN, close to the midline, innervate the HPC (Michelsen et al., 2007). In periestropausal rats, the decreased number of TPH-ir neurons in the dorsal and ventral subregions of the caudal DRN may explain the lower content of serotonin in the HPC. In accordance, estradiol treatment restored the number of TPH-ir neurons in the caudal DRN in association with the recovery of the serotonin content in the HPC. The HPC is more closely related to depression, whereas the AMY is related to anxiety (Graeff et al., 1996); suggesting a possible pathway by which estradiol therapy to perimenopausal women could be beneficial to treat depression. Accordingly, it has been shown that the administration of ER antagonists to the HPC, but not the AMY, increase anxiety and depression-like behaviors in rodents, which suggests that ERs in the HPC are a critical site for estradiol antianxiety and antidepressant-like effects (for review, see Walf and Frye, 2007). Furthermore, the antidepressant effects of estradiol on serotonergic neurotransmission and depressive behavior appear to be mediated preferentially via ERβ. For example, selective agonists for ERβ, but not ERα, produced antidepressant effects, such as decreased immobility and increased struggling and swimming in the forced swim test in rats (Walf et al., 2004; Rocha et al., 2005; Clark et al., 2012; Bansal and Chopra, 2015; Bastos et al., 2015; Benmansour et al., 2016). In addition, the antidepressant activity of estradiol has been shown to be absent in knockout mice for ERβ (Rocha et al., 2005). Moreover, recent reports have demonstrated that female mice lacking ERβ leads to dysregulation of brain-derived neurotrophic factor and serotonin signaling and decrease synaptic plasticity in the HPC, which could predispose the brain to a state of depression (Chhibber et al., 2017). Likewise, ERβ-selective ligand reduces depressive-like behavior in ovariectomized mice (Sasayama et al., 2017).

In the present study, the PR and ERβ mRNA levels in the HPC of periestropausal rats are identical to the control rats; nevertheless, estradiol therapy increased the levels of ERβ but not PR mRNA. The upregulation of ERβ expression in the DRN and HPC appears to be the mechanism by which estradiol therapy improves psychological symptoms in perimenopausal women that are not estradiol deficient. For example, it has been shown that estradiol therapy increases the serotonin turnover rate in the HPC of adult female rats (Kiss et al., 2012), and the administration of DPN, a selective agonist for ERβ, into this area decreases anxiety and depressive behaviors of ovariectomized rats (Walf and Frye, 2007). Interestingly, the intracellular effects of DPN in rat HPC neurons are similar to those obtained following treatment with sertraline, a widely used inhibitor of serotonin reuptake (Benmansour et al., 2016). Finally, it has also been shown that local application of DPN into the HPC induces slowing of 5-HT clearance, whereas an ERα agonist blocks the fluvoxamine inhibitory effect on 5-HT clearance (Benmansour et al., 2012); these findings suggest that estradiol may operate as an antidepressive through the activation of ERβ within the DRN to increase the serotonergic output to the HPC and/or directly via the activation of ERβ within the HPC.

In conclusion, our data provide novel neuroendocrine insights into the understanding of the positive effect of estradiol therapy on perimenopausal symptoms in normoestrogenic perimenopausal women. We have shown that periestropausal rats display low progesterone plasma levels and a reduced number of serotonin neurons and ERβ mRNA levels in the DRN, as well as a reduced serotonin content in the AMY and HPC. The positive effects of estradiol therapy during perimenopause appear to result, at least in part, from the increase in peripheral progesterone biosynthesis in association with an upregulation of ERβ in the DRN and dorsal HPC that appears to potentiate the DRN/dorsal HPC serotonergic circuit. Therefore, the development of new therapies to target ERβ may be an alternative to obtain the positive effects of estradiol action while eliminating the side effects of estradiol therapies that typically result from ERα activation.

## Synthesis

Reviewing Editor: Liisa Galea, University of British Columbia Decisions are customarily a result of the Reviewing Editor and the peer reviewers coming together and discussing their recommendations until a consensus is reached. When revisions are invited, a fact-based synthesis statement explaining their decision and outlining what is needed to prepare a revision will be listed below. The following reviewer(s) agreed to reveal their identity: Vibe Frokjaer, Farida Sohrabji

Reviewers read the revised manuscript and find some more changes need to be made to the manuscript.

1. There is no mention on the limitation of model in terms of non-fluctuating estradiol levels (important pathophysiological feature of perimenopause) or a mentioning of no control group with hormone/treatment exposure. This is important to mention explicitly.

2. The fact that VCD was initiated at the time of puberty (PND28 instead of PND21) which, while the authors provide some rationale, is still problematic in terms of interpretation. This nuance of initiating VCD treatment at puberty versus in adulthood should at least be discussed with caveats in the manuscript.

3. Behavioral data would certainly strengthen this manuscript. The authors state that another manuscript which includes behavior is being prepared, and this data is included in the discussion. Since there is no review of this data, including the results in the discussion is premature.

4. Estrogen is used in the significance statement - please change to estrogens or be specific.
